# Psychosocial factors associated with flourishing among Australian HIV-positive gay men

**DOI:** 10.1186/s40359-016-0154-z

**Published:** 2016-09-15

**Authors:** Anthony Lyons, Wendy Heywood, Tomas Rozbroj

**Affiliations:** Australian Research Centre in Sex, Health and Society, School of Psychology and Public Health, La Trobe University, 215 Franklin Street, Melbourne, VIC 3000 Australia

**Keywords:** HIV, Gay men, Well-being, Positive mental health, Flourishing, Positive psychology

## Abstract

**Background:**

Mental health outcomes among HIV-positive gay men are generally poorer than in the broader population. However, not all men in this population experience mental health problems. Although much is known about factors associated with depression and anxiety among HIV-positive gay men, little is known about factors associated with positive mental health. Such knowledge can be useful for optimizing well-being support programs for HIV-positive gay men.

**Methods:**

In this study, we examined flourishing, which broadly covers most aspects of positive mental health. A sample of 357 Australian HIV-positive gay men completed a survey on their mental health and well-being, including the Flourishing Scale. Given the lack of previous research, we explored a wide range of psychosocial factors, including demographics, stigma, discrimination, and social support, to identify key factors linked to flourishing.

**Results:**

The sample showed a similar level of flourishing to those in general population samples. Several independent factors were found to be associated with flourishing outcomes. Those who were most likely to be flourishing tended to have low or no internalized HIV-related stigma, were employed, received higher levels of practical support, had a sense of companionship with others, and felt supported by family.

**Conclusions:**

These and other findings presented in this article may be used to help inform strategies for promoting optimal levels of mental health, and its associated general health benefits, among HIV-positive gay men.

## Background

In high-income countries such as the United States and Australia, HIV predominately affects gay men and other men who have sex with men [1, 2]. HIV-positive gay men often face stigma and discrimination related both to being gay and having HIV. These specific stressors as well as other stress from being part of a socially devalued group can have multiple implications for health and well-being. For example, there is now substantial evidence that supports Minority Stress Theory [3], which suggests that belonging to a stigmatized group can result in additional life stress and a greater risk for mental health and other health problems [4, 5]. Identifying ways in which HIV-positive gay men may be supported to withstand or overcome the potential impact of stigma is therefore an important objective.

In this context, HIV/AIDS organizations in many countries seek to promote well-being and quality of life among people living with HIV (PLHIV). Consistent with Minority Stress Theory, a growing body of research suggests that HIV-positive gay men have much higher rates of depression and anxiety than the broader population (for example, see reviews [6–11]). However, researchers have so far focused mostly on mental illness; only a small number of studies have examined aspects of positive mental health, such as a sense of meaning, optimism, and regular experiences of positive affect. It is now well-established that a singular focus on preventing or treating mental illness does not automatically mean that an individual thrives or flourishes [12, 13]. In fact, some researchers have found that individuals can languish without experiencing a diagnosable mental illness [14].

In this article, we focus on what positive psychologists refer to as *flourishing*, which broadly covers most aspects of positive mental health [13–15]. Flourishing is conceptualized as having two main components. The first is a hedonic component in which a person experiences frequent positive emotions, such as happiness and optimism. The second is a eudemonic component in which a person has a sense of self-acceptance, a sense of meaning or purpose, and feels that much of their life is lived in accordance with their values [14, 16]. In addition to the intrinsic benefits of having good mental health, such as greater life satisfaction, there is a range of secondary health benefits. These have been well documented. For example, studies conducted in the general population have shown that having high levels of mental health are linked to lower rates of physical [17–19] and mental health problems [20]. In fact, Diener and Chan [18] reviewed seven types of evidence and found a causal association between high levels of mental health and better physical health and longevity in healthy populations.

We know of no published studies that have examined the experience of flourishing more broadly or that have used a standardized measure of flourishing among HIV-positive gay men. Some studies, however, have examined some specific aspects of flourishing. For example, a 2008 review of longitudinal studies of positive psychosocial predictors of health found optimism was associated with slower disease progression in two studies conducted after the introduction of highly active anti-retroviral therapy (HAART), and positive affect was associated with lower mortality and a better treatment response in one other study [21]. More recent studies have found optimism to be associated with greater psychological well-being and reductions in perceived HIV-related stigma among PLHIV referred for mental health services within a HIV clinic [22], while dispositional optimism and perceived confidence in one’s ability to achieve a favorable outcome were found to predict increases in positive affect over a 2-month period among participants recruited from a HIV outpatient clinic [23]. Some studies have also examined resilience among HIV-positive gay men [24], such as hardiness [25] and coping with life challenges [26]. Although resilience is an aspect of flourishing, having resilience does not necessarily mean a person is flourishing [12, 27]. Thus, studies are also needed on flourishing specifically.

The current lack of research on flourishing among HIV-positive gay men may mean that many HIV support workers, health professionals, and policymakers do not have an evidence base for devising ways of promoting mental health beyond illness prevention. To help build this evidence base, we examined flourishing in a national community-based sample of HIV-positive Australian gay men, with the specific aim of identifying a range of psychosocial and demographic factors associated with flourishing using a standard measure of flourishing.

## Methods

### Respondents

A national survey on the well-being of PLHIV was completed by 402 HIV-positive Australian residents aged 18 years or older. Nearly all identified as male (*n* = 386) and 89 % of respondents (*n* = 357) identified as gay or homosexual. Sexual identity was assessed by asking men whether they identified as ‘straight or heterosexual’, ‘gay or homosexual’, ‘bisexual’, or ‘other’. Only the responses from the 357 gay-identified men were sampled for this study.

### Survey measures

The survey items examined in this study included:

#### Outcome variable

##### Flourishing Scale

Flourishing was measured using the Flourishing Scale (FS) [28]. The FS consists of eight items assessing the main components of flourishing or social-psychological functions. Examples of items include “I lead a purposeful and meaningful life” (eudemonic component) and “I am optimistic about the future” (hedonic component). Each item is rated using a 7-point scale ranging from strongly disagree to strongly agree. Item scores are summed, with higher scores representing a greater likelihood of flourishing. In this study, the FS had an internal reliability (Cronbach’s alpha) score of 0.92.

#### Psychosocial and demographic variables

A variety of psychosocial and demographic variables were examined in the current study. We specifically focused on psychosocial variables related to stigma, discrimination, and social support. A focus on stigma and discrimination was included because previous studies have shown that stigma, whether related to HIV or sexuality, can have a powerful impact on the lives of HIV-positive gay men [24, 29]. A focus on social support was included because the provision of support is often a major focus of HIV organizations, governments, and health agencies. Social support is also linked strongly to mental health generally [30] and has been shown to be a protective factor for poorer mental health outcomes among PLHIV [31–33]. It is also one area that can be potentially modified or improved in policy and practice initiatives to help make a difference to the lives of HIV-positive gay men. Specific measures for stigma and support variables included:

##### Stigma and discrimination

According to the HIV-stigma framework, stigma can influence mental health through a number of different mechanisms [34]. In this study, we investigated two of these mechanisms; internalized HIV-related stigma (feelings and beliefs about oneself) and enacted stigma (external discrimination).

The Internalized AIDS-related stigma scale (IA-RSS) was used to measure HIV-related internalized stigma [35]. Six dichotomous items (agree versus disagree) including “It is difficult to tell people about my HIV infection” and “Being HIV positive makes me feel dirty” are used to measure shame and concealment of HIV status. Participants receive one point for every item they agree with. Higher scores indicate greater internalized stigma. In this study, internal reliability for the IA-RSS was 0.84.

Next we examined enacted stigma according to two types of discrimination, that is, HIV-related discrimination and sexuality-related discrimination. Each was assessed using separate questions; “When did you last feel like you were treated unfairly as a direct result of your HIV status / sexual orientation?” Separate variables were computed to indicate whether participants perceived themselves as having experienced each type of discrimination in the past 12 months.

##### Social support

We examined social support according to different types and sources of support. Types of social support were measured using the short-form Interpersonal Support Evaluation List (ISEL-SF) [36]. The ISEL-SF contains 12 items each rated on a 4-point scale and measures three types of social support – appraisal (emotional support), belonging (having someone to do things with), and tangible (practical help with tasks). Items are summed separately for each subscale, with higher scores indicating greater perceived social support. Internal reliability for each of the subscales in this study was 0.80 (appraisal), 0.84 (belonging), and 0.76 (tangible).

Four potential sources of social support were assessed by asking participants how much support they had received from a relationship partner, family, friends, and/or support agencies (e.g. HIV organizations, counsellors). Response options included “A lot”, “Some”, “A little”, and “None”. Participants also reported on the number of people in their life they regarded as close friends (response options were coded as 0, 1–2, 3–5, and 6 or more close friends).

##### Demographic variables

Information on the participant’s age, educational attainment, employment status, income, residential location, country of birth, and relationship status were also collected. Relationship status was assessed by asking participants whether or not they were currently in an ongoing regular relationship. Finally, participants reported the year in which they first tested HIV-positive.

### Procedure

The study was approved by the Human Ethics Committee of La Trobe University (approval number FHEC14/015). Participants were recruited via study advertisements which were distributed across multiple platforms targeting PLHIV between August and December 2014. These included Facebook, the Facebook page of The Institute of Many (a rapidly growing online community of PLHIV), Grindr (a popular dating app for gay and bisexual men), HIV organizations, and a large database of PLHIV who had participated in previous studies conducted by La Trobe University and had given their permission to be notified about future research. All advertisements directed participants to an online survey which was administered using Demographix (Demographix Limited, London) online software. Participants were informed prior to commencing the online survey that their responses were anonymous, and indicated their consent to participate before being able to proceed with the survey. No incentives were given for participating in the study. On average, the survey took 24 min to complete.

### Data analysis

Associations between flourishing and the demographic and psychosocial variables were first examined using means and unadjusted univariable linear regression models. Based on these results, variables that were associated with flourishing at *p* < 0.25 were then entered into a multiple linear regression model to identify significant independent demographic and psychosocial factors associated with flourishing. Model diagnostics, including multivariate outliers and multicollinearity were examined prior to the presentation of the final model. All associations were treated as significant at *p* < 0.05 and all analyses were conducted using Stata Version 14 (StataCorp, College Station, TX).

## Results

### Sample profile

Table 1 displays the sample profile. The majority of the 357 HIV-positive gay men who participated in the study were aged 30 years or older (92 %), lived in inner city or suburban areas (79 %), were born in Australia (79 %), had some tertiary education (79 %), and were employed full-time or part-time (70 %). Approximately one in ten (9 %) reported other types of employment, such as being self-employed or a student. Seventy-five per cent of men had first tested HIV-positive more than 5 years prior to the study being conducted (before 2010).

**Table 1:** Sample profile (*n* = 357)

	Number	Percent
Age		
18–29	30	8.5
30–49	184	52.3
50+	138	39.2
Education		
Secondary or below	76	21.4
Non-university tertiary	127	35.7
University – undergraduate	77	21.6
University – postgraduate	76	21.4
Employment		
Full time	188	53.0
Part time or casual	59	16.6
Unemployed	35	9.9
Retired	40	11.3
Other	33	9.3
Annual income (Australian dollars)		
0–19,999	61	17.6
20,000–49,999	85	24.6
50,000–99,999	133	38.4
100,000+	67	19.4
Residential location		
Inner city	209	58.7
Suburban	71	19.9
Regional/rural	76	21.4
Country of birth		
Australia	282	79.0
Overseas	75	21.0
Regular relationship		
Yes	168	47.1
No	189	52.9
Year first tested positive		
1980–1995	96	27.0
1996–2009	171	48.0
2010–2014	89	25.0
	Mean (SD)	
Flourishing (flourishing scale)	44.0 (9.4)	

### Overall flourishing scores

The mean score on the flourishing scale for this sample of HIV-positive gay men was 44.0 (SD = 9.4; median = 46; range = 12–56). We were unable to find relevant population-based data for Australia for comparing means. However, these scores were almost identical to a recent population-based study in New Zealand (male mean = 43.3, SD = 8.6, *t* (348) = 1.33, *p* = 0.18) [16]. In fact, almost 30 % of men in this sample scored at high levels on the scale (above 50, *n* = 91), equivalent to the 85^th^ percentile in the New Zealand study.

### Psychosocial and demographic factors associated with flourishing

Table 2 displays the regression results examining psychosocial and demographic factors associated with flourishing. In unadjusted analyses, higher flourishing scores were associated with greater perceived appraisal support (*F* [1, 342] = 152.58, *p* < 0.001), sense of belonging (*F* [1, 344] = 180.48, *p* < 0.001), and tangible support (*F* [1, 346] = 152.39, *p* < 0.001). Higher flourishing scores were also associated with a greater number of close friends (*F* [3, 345] = 15.81, *p* < 0.001) and being in a regular relationship (*F* [1, 347] = 6.54, *p* = 0.01). Lower flourishing scores, however, were associated with being unemployed (*F* [4, 342] = 4.75, *p* = 0.001), having a lower income (*F* [3, 334] = 3.92, *p* = 0.01), being treated unfairly because of one’s HIV status (*F* [1, 345] = 12.20, *p* < 0.001) and/or sexual orientation in the past 12 months (*F* [1, 345] = 7.38, *p* = 0.01), greater internalized stigma (*F* [1, 342] = 32.41, *p* < 0.001), and receiving limited support from partners (*F* [3, 345] = 6.49, *p* < 0.001), friends (*F* [3, 345] = 40.23, *p* < 0.001), or family (*F* [3, 345] = 18.91, *p* < 0.001).

**Table 2:** Regression results for scores on the Flourishing Scale (*n* = 349)

		Unadjusted				Adjusted^b^			
	Mean	B	(SE B)	β		B	(SE B)	β	
Age					*p* = 0.45				
18–29	43.3	-0.19	(1.87)	-0.01					
30–49^a^	43.5	-	-	-					
50+	44.8	1.31	(1.08)	0.07					
Education					*p* = 0.11				*p* = 0.31
Secondary or below^a^	42.0	-	-	-		-	-	-	
Non-university tertiary	43.6	1.69	(1.38)	0.09		0.06	(1.06)	-0.003	
University – undergraduate	45.0	3.01	(1.54)	0.13		1.57	(1.19)	0.07	
University – postgraduate	45.4	3.46	(1.54)	0.15*		1.53	(1.21)	0.07	
Employment					*p* = 0.001				*p* = 0.01
Full time^a^	45.1	-	-	-		-	-	-	
Part time or casual	44.3	-0.84	(1.41)	-0.03		1.34	(1.26)	0.06	
Unemployed	38.1	-7.05	(1.75)	-0.22**		-4.41	(1.77)	-0.14*	
Retired	44.9	-0.16	(1.63)	-0.01		0.97	(1.60)	0.03	
Other	41.5	-3.56	(1.75)	-0.11*		0.21	(1.71)	0.01	
Income (Australian dollars)					*p* = 0.009				*p* = 0.07
0–19,999	41.1	-3.85	(1.47)	-0.15**		-2.41	(1.53)	-0.10	
20,000–49,999	42.9	-1.99	(1.31)	-0.09		-2.15	(1.26)	-0.10	
50,000–99,999^a^	44.9	-	-	-		-	-	-	
100,000+	46.2	1.28	(1.41)	0.05		1.67	(1.09)	0.07	
Residential location					*p* = 0.24				*p* = 0.21
Inner city^a^	43.9	-	-	-		-	-	-	
Suburban	42.7	-1.15	(1.32)	-0.05		0.85	(1.00)	0.04	
Regional/rural	45.4	1.52	(1.29)	0.07		1.72	(1.00)	0.08	
Country of birth					*p* = 0.19				*p* = 0.89
Australia^a^	43.6	-	-	-		-	-	-	
Overseas	45.2	1.61	(1.24)	0.07		0.13	(0.96)	0.01	
Regular relationship^c^					*p* = 0.01				
Yes	45.4	2.57	(1.01)	0.14*					
No^a^	42.8	-	-	-					
Year first tested positive					*p* = 0.82				
1980–1995	43.7	-0.62	(1.22)	-0.03					
1996–2009^a^	44.3	-	-	-					
2010–2014	43.6	-0.66	(1.25)	-0.03					
Perceived discrimination due to HIV status past 12 m					*p* < 0.001				*p* = 0.36
Yes	41.0	-4.01	(1.15)	-0.18**		0.96	(1.04)	0.05	
No^a^	45.0	-	-	-		-	-	-	
Perceived discrimination due to sexual orientation past 12 m					*p* = 0.01				*p* = 0.65
Yes	41.1	-3.54	(1.30)	-0.14**		-0.50	(1.11)	-0.02	
No^a^	44.7	-	-	-		-	-	-	
Internalized AIDS-related Stigma Scale		-1.29	(0.23)	-0.29**	*p* < 0.001	-0.50	(0.20)	-0.12*	*p* = 0.01
Number of close friends					*p* < 0.001				*p* = 0.30
0^a^	42.8	-	-	-		-	-	-	
1–2	38.4	-4.42	(1.64)	-0.17**		-1.56	(1.34)	-0.06	
3–5	43.6	0.84	(1.34)	0.04		-0.97	(1.14)	-0.05	
6+	48.5	5.69	(1.43)	0.27**		0.60	(1.22)	0.03	
Social support - appraisal		1.68	(0.14)	0.56**	*p* < 0.001	0.28	(0.21)	0.10	*p* = 0.18
Social support - belonging		1.68	(0.12)	0.59**	*p* < 0.001	0.64	(0.19)	0.23**	*p* = 0.001
Social support - tangible		1.73	(0.14)	0.55**	*p* < 0.001	0.44	(0.22)	0.15*	*p* = 0.05
Support from partner/spouse					*p* < 0.001				*p* = 0.49
A lot^a^	47.0	-	-	-		-	-	-	
Some	43.1	-3.89	(1.73)	-0.13*		-1.40	(1.36)	-0.05	
A little	42.2	-4.77	(3.04)	-0.08		2.14	(2.36)	0.04	
None	42.3	-4.69	(1.08)	-0.25**		-0.08	(0.92)	-0.004	
Support from friends					*p* < 0.001				*p* = 0.13
A lot^a^	48.3	-	-	-		-	-	-	
Some	44.8	-3.46	(1.01)	-0.18**		0.38	(0.99)	0.02	
A little	37.4	-10.89	(1.18)	-0.47**		-1.42	(1.35)	-0.06	
None	31.8	-16.46	(2.22)	-0.35**		-4.70	(2.45)	-0.10	
Support from family					*p* < 0.001				*p* = 0.03
A lot^a^	49.2	-	-	-		-	-	-	
Some	44.8	-4.43	(1.27)	-0.22**		-1.35	(1.08)	-0.07	
A little	40.5	-8.71	(1.33)	-0.40**		-3.05	(1.22)	-0.15*	
None	40.4	-8.80	(1.42)	-0.37**		-3.32	(1.28)	-0.14*	
Support from agencies					*p* = 0.13				*p* = 0.33
A lot^a^	47.0	-	-	-		-	-	-	
Some	44.4	-2.57	(1.84)	-0.11		-0.54	(1.43)	-0.02	
A little	42.9	-4.07	(1.80)	-0.18*		-2.21	(1.37)	-0.10	
None	43.6	-3.38	(1.66)	-0.18*		-1.20	(1.28)	-0.07	

*R* ^2^ = 0.55, adjusted *R* ^2^ = 0.50, F(34, 281) = 10.13, *p* < 0.001

^a^Reference category

^b^Adjusted for education, employment, income, residential location, country of birth, treated unfairly because of HIV status in past 12 m, treated unfairly because of sexual orientation past 12 m, internalized AIDS-related stigma, number of close friends, appraisal support, belonging support, tangible support, support from partner/spouse, support from friends, support from family, and support from agencies

^c^Not included in multivariate model due to co-linearity issues with support from partner

*p < 0.05; **p < 0.01

B = unstandardized beta coefficient; SE = standard error; β = standardized beta coefficient

A multivariable linear regression was conducted to identify significant independent psychosocial and demographic factors. Following an examination of model diagnostics, multivariate outliers (*n* = 3) were removed to improve the normality of residuals and model fit. Multicollinearity between the psychosocial variables was also examined. All variance inflation values (VIF) were below 5 and tolerance scores were above 0.2 indicating no problems [37]. After adjusting for the other psychosocial demographic variables entered into the regression, significant independent factors associated with higher flourishing scores included a greater perceived sense of belonging (*F* [1, 281] = 11.58, *p* < 0.001) and tangible support (*F* [1, 281] = 3.92, *p* = 0.05). Significant independent factors associated with lower flourishing scores included receiving little or no support from family (*F* [3, 281] = 2.93, *p* = 0.04), experiencing greater internalized stigma (*F* [1, 281] = 6.01, *p* = 0.01), and being unemployed (*F* [4, 281] = 3.35, *p* = 0.01). Variables no longer associated with flourishing after adjustments included, unfair treatment due to one’s HIV status or sexual orientation in the past 12 months, number of close friends, appraisal social support, and support from partners or friends. Overall model fit was adjusted-*R*^*2*^ = 0.50, indicating one half of the variance in flourishing scores was predicted by variables in the model.

## Discussion

In this study, we examined aspects of positive mental health in a national community-based sample of Australian HIV-positive gay men. Overall, this sample demonstrated a similar level of flourishing compared to at least one set of general population-based norms for the FS. Although our sample was large, it was not population-based and therefore needs to be treated with some caution with regard to representativeness. However, our study perhaps gives an initial indication that although a large number of Australian HIV-positive gay men may be struggling with regard to their mental health, as has been shown in other studies [29], many are also doing well. Further learning about the lives of those who are doing well may therefore be beneficial to understanding how to support those who are facing mental health challenges.

To assist with this, we examined psychosocial and demographic factors associated with flourishing. One strong factor was internalized HIV-related stigma. Greater levels of internalized stigma were associated with lower levels of flourishing. Thus, it appears from these findings that stigma may be a major factor not just in mental health problems [29] but also as a possible barrier to flourishing. Eradicating stigma is currently a major goal of international efforts to prevent HIV, such as the “90:90:90” initiative [38]. However, challenging the public attitudes and beliefs that give rise to stigma can be a lengthy process [39, 40]. In the meantime, focusing on further ways to help HIV-positive gay men minimize the psychological impact of stigma may also be needed. Fostering support networks is perhaps one potential way to help buffer stigma-related stress [41]. Peer-led counseling and support is one option that may help to improve well-being among PLHIV [42]. Individual-based support programs may also be helpful. For example, there has been considerable growth in self-directed online interventions that step users through personal growth programs, such as those based on cognitive behavioral therapy (CBT) [43]. However, tailored programs that specifically address experiences of living with HIV are needed and may be an area worth exploring for future development and research.

Employment is another potential area of focus. Employment status was significantly associated with flourishing. Specifically, unemployed HIV-positive gay men reported lower levels of flourishing. These findings are consistent with previous studies showing better physical and mental health outcomes among PLHIV who have regular employment [44–47]. These health benefits are likely related to the structure, social support, and meaning provided by being employed [44, 45], although the exact nature and direction of the relationship between employment and mental health is not known [44, 46, 47]. Longitudinal studies are needed to examine the degree to which employment leads to better mental health outcomes or whether better mental health helps PLHIV maintain ongoing employment. A number of factors, however, can make obtaining and retaining employment difficult for PLHIV, including stigma, confidentiality, disclosure, and the ability to take time off during any periods of poor health or to attend medical appointments [48]. HIV support agencies could perhaps consider career counseling and employment support for those seeking work. This recommendation echoes previous research where PLHIV in Australia have identified issues related to work or employment as one of the main areas where they lack information [48].

Encouragingly, after adjustments, other socioeconomic variables such as education, income, and residential location were not significant barriers to flourishing. The lack of a significant association between income and flourishing is particularly noteworthy. Previous studies of HIV-positive gay men have tended to show strong links between income and mental health [49]. However, studies have generally focused on mental health problems, such as depression, or well-being more generally. Achieving optimal levels of mental health often requires a targeted approach, with a focus on factors not necessarily linked with mental illness [13]. Indeed, some studies of the general population have shown that income and wealth may be protective of lower levels of well-being but not necessarily promotive of higher levels [50]. Although further investigation would be needed, it appears thus far from our study that income is likewise less important than other factors, such as employment, in whether HIV-positive gay men experience positive mental health.

One further important way in which strategies aimed at promoting positive well-being may need to differ from those targeting mental illness is in relation to social support. Specifically, having access to practical or material support (measured as tangible support) and companionship or having someone to do things with (measured as belonging support) were linked to higher levels of flourishing. These findings differ from previous studies showing a strong link between (lack of access to) emotional support and poorer mental health outcomes among a sample of older Australian gay men [51] and a sample of HIV-positive gay men [29]. It may be that emotional support is mostly beneficial when a person is experiencing challenges and therefore serves primarily as a protective role. When relatively healthy, tangible support and a sense of belonging or companionship may be more important to a person’s well-being or sense of feeling supported in life. This, however, is largely speculative. Further research is needed to fully understand how particular types of support might be important to flourishing. For now, it would appear at least from our study that some types of support may be more conducive to flourishing than other types, and this might therefore need to be considered in efforts toward promoting higher levels of mental health among HIV-positive gay men.

After adjusting for other psychosocial variables, support from family members was also independently associated with higher levels of flourishing. It is well known that some men have experienced rejection or a loss of support from family due to being gay and/or having HIV [52, 53], which is likely to have a deep psychological impact for many of those affected. It would thus appear that a lack of family support serves as a potential barrier to flourishing. Addressing ways in which this challenge might be overcome is another possible consideration for any initiatives aimed at promoting optimal levels of mental health among HIV-positive gay men. Where possible, initiatives might therefore consider the provision of family-based counseling or other interventions to improve family support for HIV-positive gay men [54]. Health professionals and support workers might also consider reaching out to the family members of their HIV-positive clients to offer education or advice. Assisting men to improve their relationships with family might further prove useful in some cases. Where stigma is a particular issue, public stigma-reduction programs that address community attitudes toward HIV-positive gay men may also be beneficial for helping families cope and respond more positively toward family members who are either gay or living with HIV, especially if families fear being stigmatized themselves [55].

The broader health benefits of promoting higher levels of mental health and positive well-being have been documented in both the general population [18] and among PLHIV [21]. As noted earlier, high levels of mental health are likely to lead to better physical health. A good case therefore exists for HIV organizations, support workers, health professionals, and policymakers to consider ways of not only preventing mental health problems among HIV-positive gay men, but also fostering higher levels of mental health. A range of studies has now demonstrated that higher levels of mental health can be increased through positive psychological interventions such as individual therapy, group training, and self-help interventions [56, 57]. There may also be scope for providing interventions or support programs via the web or smartphone apps [58]. We know of no interventions currently that specifically aim to promote flourishing among HIV-positive gay men, so this is an area that may well deserve attention in the future.

This was one of the first studies to examine psychosocial factors associated with flourishing among HIV-positive gay men. An important strength of the study was having collected a national sample. Participants were recruited from a diverse range of backgrounds and residential locations across Australia. However, being community-based, we do not know for sure whether it was representative of all HIV-positive gay men and additional studies would be needed to further corroborate our findings. We did however use a number of different strategies to increase the diversity of participants. Facebook was the most successful strategy (56 %), followed by email advertisements (13 %) and HIV organizations (9 %). However, as the sample was recruited online, response rates cannot be calculated as we do not know how many potential participants saw the advertisements. Unfortunately, representative data are not available for Australian HIV-positive gay men, so it is not possible to assess the representativeness of any study at this time. The demographic characteristics of our sample are, however, similar to other Australian national studies of PLHIV [48]. Nevertheless, our sample was relatively large and diverse, which covered all major demographics, including a range of socioeconomic backgrounds. The study was also cross-sectional, so it is not possible to identify directions of causality between variables. For example, it may be possible that individuals who are flourishing simply find it easier to gain support from others. That said, studies do show that social support brings benefits to mental health [30, 32, 33], so it is likely that social support also helps to facilitate flourishing. Even so, further research is recommended that draws upon longitudinal data to fully identify directions of causality.

Our findings were further limited to HIV-positive gay men due to small numbers of women and heterosexual men completing the survey, a finding that reflects the prevalence of HIV in the Australian population [48]. It is therefore unknown whether these findings can be generalized to other PLHIV subpopulations. Furthermore, to contextualize our findings on flourishing, we compared overall means for the sample with a general population study conducted in New Zealand. This is the only population-based study we were able to find that had used the FS and therefore reported population norms. No such Australian data is available. Our comparisons should therefore be treated with caution. Although New Zealand is culturally and economically similar to Australia, a more reliable comparison can only be conducted when Australian population data on the FS becomes available.

## Conclusion

This is among the first studies of flourishing among HIV-positive gay men. In this national sample of Australian HIV-positive gay men, internalized HIV-related stigma was found to be a major barrier to flourishing. Higher levels of flourishing, however, were found among those who perceived a greater level of practical support in their lives, who had a sense of belonging or companionship, and who felt supported by family. These findings provide guidance for policymakers, health professionals, support workers, and anyone seeking to optimize support programs for HIV-positive gay men, with internalized HIV-related stigma and specific aspects of social support likely to require attention. In particular, this study and its findings offer new information to help facilitate programs that are not only aimed at treating or preventing mental illness among HIV-positive gay men, but also seek to foster higher levels of well-being or indeed flourishing.

## Abbreviations

FS: Flourishing scale
HAART: Highly active anti-retroviral therapy
HIV: Human immunodeficiency virus
IA-RSS: Internalized AIDS-related stigma scale
ISEL-SF: Interpersonal Support Evaluation List - short-form
PLHIV: People living with HIV

## References

[1] Centers for Disease Control and Prevention: HIV Surveillance Report, 2014, vol. 26. http://www.cdc.gov/hiv/library/reports/surveillance/. Published November 2015. Access August 2016.

[2] The Kirby Institute (2015). HIV, viral hepatitis and sexually transmissible infections in Australia Annual Surveillance Report 2015.

[3] Meyer IH (2003). Prejudice, social stress, and mental health in lesbian, gay, and bisexual populations: Conceptual issues and research evidence. Psychol Bull.

[4] Hatzenbuehler ML, McLaughlin KA, Keyes KM, Hasin DS (2010). The impact of institutional discrimination on psychiatric disorders in lesbian, gay, and bisexual populations: a prospective study. Am J Public Health.

[5] Hatzenbuehler ML, Phelan JC, Link BG (2013). Stigma as a fundamental cause of population health inequalities. Am J Public Health.

[6] Arseniou S, Arvaniti A, Samakouri M (2014). HIV infection and depression. Psychiatry Clin Neurosci.

[7] Ciesla JA, Roberts JE (2001). Meta-analysis of the relationship between HIV infection and risk for depressive disorders. Am J Psychiatry.

[8] Rabkin JG (2008). HIV and depression: 2008 review and update. Curr HIV/AIDS Rep.

[9] Sherr L, Clucas C, Harding R, Sibley E, Catalan J (2011). HIV and Depression – a systematic review of interventions. Psychol Health Med.

[10] Simoni JM, Safren SA, Manhart LE, Lyda K, Grossman CI, Rao D, Mimiaga MJ, Wong FY, Catz SL, Blank MB (2011). Challenges in addressing depression in HIV research: assessment, cultural context, and methods. AIDS Behav.

[11] Springer SA, Dushaj A, Azar MM (2012). The impact of DSM-IV mental disorders on adherence to combination antiretroviral therapy among adult persons living with HIV/AIDS: a systematic review. AIDS Behav.

[12] Keyes CL (2005). Mental illness and/or mental health? Investigating axioms of the complete state model of health. J Consult Clin Psychol.

[13] Keyes CL (2007). Promoting and protecting mental health as flourishing: a complementary strategy for improving national mental health. Am Psychol.

[14] Keyes CL (2002). The mental health continuum: from languishing to flourishing in life. J Health Soc Behav.

[15] Keyes CLM, Weiner IB, Craighead WE (2010). Flourishing. The Corsini Encyclopedia of Psychology.

[16] Hone L, Jarden A, Schofield G (2014). Psychometric properties of the Flourishing Scale in a New Zealand sample. Soc Indic Res.

[17] Chida Y, Steptoe A (2008). Positive psychological well-being and mortality: a quantitative review of prospective observational studies. Psychosom Med.

[18] Diener E, Chan MY (2011). Happy people live longer: subjective well‐being contributes to health and longevity. Appl Psychol Health Well Being.

[19] Siahpush M, Spittal M, Singh GK (2008). Happiness and life satisfaction prospectively predict self-rated health, physical health, and the presence of limiting, long-term health conditions. Am J Health Promot.

[20] Keyes CL, Dhingra SS, Simoes EJ (2010). Change in level of positive mental health as a predictor of future risk of mental illness. Am J Public Health.

[21] Ironson GH, Hayward H (2008). Do positive psychosocial factors predict disease progression in HIV-1? A review of the evidence. Psychosom Med.

[22] Ammirati RJ, Lamis DA, Campos PE, Farber EW (2015). Optimism, well-being, and perceived stigma in individuals living with HIV. AIDS Care.

[23] Mukolo A, Wallston KA (2012). The relationship between positive psychological attributes and psychological well-being in persons with HIV/AIDS. AIDS Behav.

[24] Lyons A, Heywood W, Rozbroj T (2016). Psychosocial factors associated with resilience in a national community-based cohort of Australian gay men living with HIV. AIDS Behav.

[25] Farber EW, Schwartz JAJ, Schaper PE, Moonen DJ, McDaniel JS (2000). Resilience factors associated with adaptation to HIV disease. Psychosomatics.

[26] Fang X, Vincent W, Calabrese SK, Heckman TG, Sikkema KJ, Humphries DL, Hansen NB (2015). Resilience, stress, and life quality in older adults living with HIV/AIDS. Aging Ment Health.

[27] Ryff CD, Singer B, Keyes CLM, Haidt J (2003). Flourishing under fire: Resilience as a prototype of challenged thriving. Flourishing: Positive psychology and the life well-lived.

[28] Diener E, Wirtz D, Tov W, Kim-Prieto C, Choi D-w, Oishi S, Biswas-Diener R (2010). New well-being measures: short scales to assess flourishing and positive and negative feelings. Soc Indic Res.

[29] Heywood W, Lyons A (2016). HIV and elevated mental health problems: diagnostic, treatment, and risk patterns for depression, anxiety, and stress in a national community-based cohort of gay men living with HIV. AIDS Behav.

[30] Kawachi I, Berkman LF (2001). Social ties and mental health. J Urban Health.

[31] Hall VP (1999). The relationship between social support and health in gay men with HIV/AIDS: an integrative review. J Assoc Nurses AIDS Care.

[32] Lam PK, Naar-King S, Wright K (2007). Social support and disclosure as predictors of mental health in HIV-positive youth. AIDS Patient Care STDs.

[33] McDowell TL, Serovich JM (2007). The effect of perceived and actual social support on the mental health of HIV-positive persons. AIDS Care.

[34] Earnshaw VA, Chaudoir SR (2009). From conceptualizing to measuring HIV stigma: a review of HIV stigma mechanism measures. AIDS Behav.

[35] Kalichman SC, Simbayi LC, Cloete A, Mthembu PP, Mkhonta RN, Ginindza T (2009). Measuring AIDS stigmas in people living with HIV/AIDS: the internalized AIDS-related stigma scale. AIDS Care.

[36] Cohen S, Mermelstein R, Kamarck T, Hoberman HM, Sasason IG, Sarason B (1985). Measuring the functional components of social support. Social support: Theory, research and applications.

[37] O’Brien RM (2007). A caution regarding rules of thumb for variance inflation factors. Qual Quant.

[38] Joint United Nations Programme on HIV/AIDS: 90-90-90: An ambitious treatment target to help end the AIDS epidemic. Geneva: UNAIDS; 2014.

[39] Lyons A, Kashima Y (2001). The reproduction of culture: communication processes tend to maintain cultural stereotypes. Soc Cogn.

[40] Lyons A, Kashima Y (2003). How are stereotypes maintained through communication? The influence of stereotype sharedness. J Pers Soc Psychol.

[41] Lyons A, Heywood W. Collective resilience as a protective factor for the mental health and well-being of HIV-positive gay men. Psychology of Sexual Orientation and Gender Diversity. 2016;In press.

[42] Harris GE, Larsen D (2007). HIV peer counseling and the development of hope: perspectives from peer counselors and peer counseling recipients. AIDS Patient Care STDs.

[43] Rozbroj T, Lyons A, Pitts M, Mitchell A, Christensen H (2014). Assessing the applicability of e-therapies for depression, anxiety, and other mood disorders among lesbians and gay men: analysis of 24 web-and mobile phone-based self-help interventions. J Med Internet Res.

[44] Degroote S, Vogelaers D, Vandijck DM (2014). What determines health-related quality of life among people living with HIV: an updated review of the literature. Arch Public Health.

[45] Hoffman MA (1997). HIV disease and work: effect on the individual, workplace, and interpersonal contexts. J Vocat Behav.

[46] Rueda S, Raboud J, Mustard C, Bayoumi A, Lavis JN, Rourke SB (2011). Employment status is associated with both physical and mental health quality of life in people living with HIV. AIDS Care.

[47] Worthington C, Krentz H (2005). Socio-economic factors and health-related quality of life in adults living with HIV. Int J STD AIDS.

[48] Grierson JW, Pitts M, Koelmeyer R (2013). HIV Futures Seven: The Health and Wellbeing of HIV Positive People in Australia, monograph series number 88.

[49] Lyons A, Pitts M, Grierson J (2012). Exploring the psychological impact of HIV: health comparisons of older Australian HIV-positive and HIV-negative gay men. AIDS Behav.

[50] Kahneman D, Deaton A (2010). High income improves evaluation of life but not emotional well-being. Proc Natl Acad Sci.

[51] Lyons A (2016). Social support and the mental health of older gay men: findings from a national community-based survey. Res Aging.

[52] Barrett C, Whyte C, Comfort J, Lyons A, Crameri P (2015). Social connection, relationships and older lesbian and gay people. Sex Relation Ther.

[53] Laryea M, Gien L (1993). The Impact of HIV-positive diagnosis on the individual, part 1 stigma, rejection, and loneliness. Clin Nurs Res.

[54] Rotheram-Borus M, Flannery D, Rice E, Lester P (2005). Families living with HIV. AIDS Care.

[55] Bogart LM, Cowgill BO, Kennedy D, Ryan G, Murphy DA, Elijah J, Schuster MA (2008). HIV-related stigma among people with HIV and their families: a qualitative analysis. AIDS Behav.

[56] Bolier L, Haverman M, Westerhof GJ, Riper H, Smit F, Bohlmeijer E (2013). Positive psychology interventions: a meta-analysis of randomized controlled studies. BMC Public Health.

[57] Sin NL, Lyubomirsky S (2009). Enhancing well-being and alleviating depressive symptoms with positive psychology interventions: a practice-friendly meta-analysis. J Clin Psychol.

[58] Lyons A, Rozbroj T, Pitts M, Mitchell A, Christensen H (2015). Improving E-therapy for Mood Disorders Among Lesbians and Gay Men: A Practical Toolkit for Developing Tailored Web and Mobile Phone-based Depression and Anxiety Interventions. Monograph series No. 102.

